# Eligibility of cardiac arrest patients for extracorporeal cardiopulmonary resuscitation and their clinical characteristics: a retrospective two-centre study

**DOI:** 10.1097/MEJ.0000000000001092

**Published:** 2023-10-06

**Authors:** Rob J.C.G. Verdonschot, Floor I. Buissant des Amorie, Seppe S.H.A. Koopman, Wim J.R. Rietdijk, Sindy Y. Ko, Upasna R.U. Sharma, Marc Schluep, Corstiaan A. den Uil, Dinis dos Reis Miranda, Loes Mandigers

**Affiliations:** a Emergency Department, Erasmus Medical Center; b Department of Anesthesiology, Maasstad Hospital; c Department of Hospital Pharmacy, Erasmus University Medical Center, Rotterdam; d Chief Data Office, Department of Institutional Affairs, Vrije Universiteit, Amsterdam; e Department of Anesthesiology, Erasmus Medical Center, Rotterdam; f Department of Anesthesiology and Intensive Care, Bravis Hospital, Bergen op Zoom; g Department of Intensive Care, Erasmus Medical Center; h Department of Cardiology, Erasmus University Medical Center; i Department of Intensive Care, Maasstad Hospital, Rotterdam; j Department of Cardiology, Catharina Hospital, Eindhoven, the Netherlands

**Keywords:** cardiac arrest, extracorporeal cardiopulmonary resuscitation, in-hospital cardiac arrest, mortality, out-of-hospital cardiac arrest

## Abstract

**Background and importance:**

Sudden cardiac arrest has a high incidence and often leads to death. A treatment option that might improve the outcomes in refractory cardiac arrest is Extracorporeal Cardiopulmonary Resuscitation (ECPR).

**Objectives:**

This study investigates the number of in-hospital cardiac arrest (IHCA) and out-of-hospital cardiac arrest (OHCA) patients eligible to ECPR and identifies clinical characteristics that may help to identify which patients benefit the most from ECPR.

**Design, settings and participants:**

A retrospective two-centre study was conducted in Rotterdam, the Netherlands. All IHCA and OHCA patients between 1 January 2017 and 1 January 2020 were screened for eligibility to ECPR. The primary outcome was the percentage of patients eligible to ECPR and patients treated with ECPR. The secondary outcome was the comparison of the clinical characteristics and outcomes of patients eligible to ECPR treated with conventional Cardiopulmonary Resuscitation (CCPR) vs. those of patients treated with ECPR.

**Main results:**

Out of 1246 included patients, 412 were IHCA patients and 834 were OHCA patients. Of the IHCA patients, 41 (10.0%) were eligible to ECPR, of whom 20 (48.8%) patients were actually treated with ECPR. Of the OHCA patients, 83 (9.6%) were eligible to ECPR, of whom 23 (27.7%) were actually treated with ECPR. In the group IHCA patients eligible to ECPR, no statistically significant difference in survival was found between patients treated with CCPR and patients treated with ECPR (hospital survival 19.0% vs. 15.0% respectively, 4.0% survival difference 95% confidence interval −21.3 to 28.7%). In the group OHCA patients eligible to ECPR, no statistically significant difference in-hospital survival was found between patients treated with CCPR and patients treated with ECPR (13.3% vs. 21.7% respectively, 8.4% survival difference 95% confidence interval −30.3 to 10.2%).

**Conclusion:**

This retrospective study shows that around 10% of cardiac arrest patients are eligible to ECPR. Less than half of these patients eligible to ECPR were actually treated with ECPR in both IHCA and OHCA.

## Introduction

Despite ongoing research, survival with favourable neurologic outcome after sudden cardiac arrest remains poor. Short-term survival after in-hospital cardiac arrest (IHCA) varies between 15 and 34% [1,2]. The pooled one-year survival for IHCA patients is 13% [3]. Of these survivors, 92% had moderate to good neurological performance [3]. Short-term survival after out-of-hospital cardiac arrest (OHCA) varies between 1% and 31%, with survival rates of 6–31% in the Netherlands in particular [4]. Neurologically favourable survival after OHCA is around 8% [5–7].

A longer delay to the return of spontaneous circulation (ROSC) is correlated with poor survival rates [8]. To improve the outcomes in refractory cardiac arrest patients, Extracorporeal Cardiopulmonary Resuscitation (ECPR) can be used.

Survival rates of IHCA patients treated with ECPR vary between 11 and 33% [9–11]. However, the number of studies reporting survival rates and neurological outcomes after ECPR in IHCA patients are limited. For OHCA patients, survival rates after ECPR are between 8 and 37% [9–12]. There is ongoing debate on the effects of ECPR on survival in OHCA patients, some studies suggest a survival benefit [13–15]. However, there are studies suggesting no difference in survival [16].

Previous research has reported that around 10% of IHCA and 10% of OHCA patients are eligible to ECPR [17–20]. However, the proportion of patients eligible to ECPR in the Netherlands has not been investigated. In the Netherlands, a nationwide response system exists that alerts trained citizens when an OHCA occurs in their neighbourhood [21]. This enables citizens to perform basic life support and connect the Automatic External Defibrillator (AED) before arrival of the Emergency Medical Services (EMS) [21]. As a result, the AED is more frequently used in the Netherlands (29–65%) than in other countries (2.1–3.8%) [22–24]. Furthermore, the Netherlands is a small and densely populated country with a short travel time to the hospital [25].

The high use of AEDs and the short travel time to the hospital in the Netherlands could increase the percentage of OHCA patients eligible to ECPR [22–25]. Therefore, we hypothesised that in the Netherlands more cardiac arrest patients are eligible to ECPR compared to countries with longer EMS travel times. This study aims to investigate the percentage of IHCA and OHCA patients eligible to ECPR, and to analyse their clinical characteristics.

## Methods

### Study design

This retrospective cohort study was conducted in two primary percutaneous coronary intervention (PCI) centres in Rotterdam, the Netherlands: the Erasmus University Medical Center (EMC) and the Maasstad Hospital (MSZ). Both hospitals are located in the EMS region ‘Rotterdam Rijnmond’. The EMC provides ECPR services in increasing numbers of cardiac arrest patients. The MSZ does not provide ECPR services. The Medical Ethics Committees of the EMC and MSZ approved this study (number MEC-2019-0076) and the need for informed consent was waived. Information about the prehospital setting is described in Supplementary Material, Appendix A, Supplemental digital content 1, http://links.lww.com/EJEM/A405.

### Study population

All adult (≥18 years) IHCA and OHCA patients treated in the EMC or MSZ, in the pre-COVID-19 period between 1 January 2017 and 1 January 2020 were screened. These patients were identified retrospectively by searching the electronic patient files on cardiopulmonary resuscitation. Patients with a traumatic cardiac arrest or death before arrival at the emergency department (ED) were excluded from this study. All cardiac arrest patients were retrospectively screened for ECPR eligibility using the inclusion criteria of the EMC ECPR protocol. The inclusion criteria according to the local protocol were: age ≤ 70 years, no ROSC before arrival at the ED for OHCA patients, witnessed arrest or signs of life within 5 min (e.g. movement or breathing), no-flow duration of ≤ 5 min, CPR duration ≥ 20 min, activities of daily living (ADL) independent before cardiac arrest. The exclusion criteria were: do not resuscitate (DNR) order, end-tidal CO_2_ (etCO_2_) < 9.75 mmHg after 20 min of CPR, known active malignancy, haemorrhagic shock, known liver disease with Model for End-stage Liver Disease score >30 or with use of Terlipressin, known intracranial surgery/cerebrovascular accident (CVA) within 6 weeks, mediastinitis with sternum removal, cardiac arrest based on asystole ≥20 min, known severe peripheral arterial disease (defined as signs of ischaemia or femoral artery not visible on ultrasound), expected CPR duration until start of ECPR cannulation >60 min. Patients who were eligible to ECPR, either treated with conventional Cardiopulmonary Resuscitation (CCPR) or ECPR, were included in the analysis. See the Supplementary Material for more information about the ECPR routine process and the ECPR procedure (Appendix B and C, Supplemental digital content 1, http://links.lww.com/EJEM/A405).

### Primary and secondary outcomes

The primary outcome was the proportion of patients in need of CPR who could be treated with ECPR. The secondary outcomes were ED survival, ICU survival, hospital survival, maximum Glasgow Coma Scale (GCS) score, and regain of consciousness (defined as a Glasgow motor score of 6).

### Definition of variables

The following patient characteristics were extracted from the patient records: sex, age, medical history, cardiac risk factors (diabetes mellitus, hypercholesterolaemia, high blood pressure, smoking, family history of cardiovascular disease, and obesity), and alcohol and drug abuse. The following clinical characteristics were extracted: cardiac arrest characteristics [witnessed arrest, bystander CPR, no-flow time, low-flow time, mechanical CPR, primary cardiac rhythm, and automated external defibrillator (AED) connected], administered CPR medication, primary cardiac rhythm, end-tidal CO_2_, cause of arrest (arrythmia, coronary artery disease, pulmonary embolism, intoxication, tamponade, tension pneumothorax, hypoxaemia, hypovolemia, hypothermia, hypokalaemia or hyperkaliaemia, and neurological events [e.g. intracranial bleeding or ischemic events]), treatment (internal cardiac defibrillator, PCI, intra-aortic balloon pump, coronary artery bypass graft, impella, veno-arterial extracorporeal membrane oxygenation placed during hospital stay, left-ventricular assist device and inotropes/vasopressors), complications [acute kidney injury (AKI), CVA, re-arrest, infectious complications, liver failure, delirium, post-anoxic brain injury, pulmonary embolism, and bleeding] and laboratory values at admission.

The following outcome variables were extracted: ED survival (patients who survived ED admission and were transported to the ICU or the ward), ICU survival (patients who survived ICU admission and were transported to the ward or home), hospital survival, return of circulation (ROC, that is, ROSC for CCPR patients and starting ECMO flow in ECPR patients), length of hospital stay, regain of consciousness, maximum GCS score, and cause of death.

### Statistical analysis

The data was analysed in three steps. First, the characteristics of the patients were described. Continuous variables were reported as medians and interquartile ranges (IQR). Categorical variables were reported as numbers and percentages. The Kruskal–Wallis test was used for examining differences between two groups for continuous variables. The Fisher’s exact test was used to compare distributions of categorical data. Second, the primary outcome was estimated, namely the total number of cardiac arrest patients who were eligible to ECPR. We compared this to the number of patients that were actually treated with ECPR. Third, we compared the clinical characteristics of patients eligible to ECPR who were treated with CCPR with the clinical characteristics of ECPR patients, using similar statistical methods as in the first step. IHCA and OHCA patients differ in several cardiac arrest characteristics. As these differences could influence the ECPR eligibility as well as the outcomes, these groups were analysed separately. For data management, IBM SPSS Statistics 26 was used. Statistical analyses were performed using R studio (version 6.3). A p-value of < 0.05 was considered statistically significant.

## Results

In total, 1246 patients were included, of whom 412 were IHCA patients and 834 were OHCA patients.

### Primary outcome IHCA patients

**Fig. 1:**
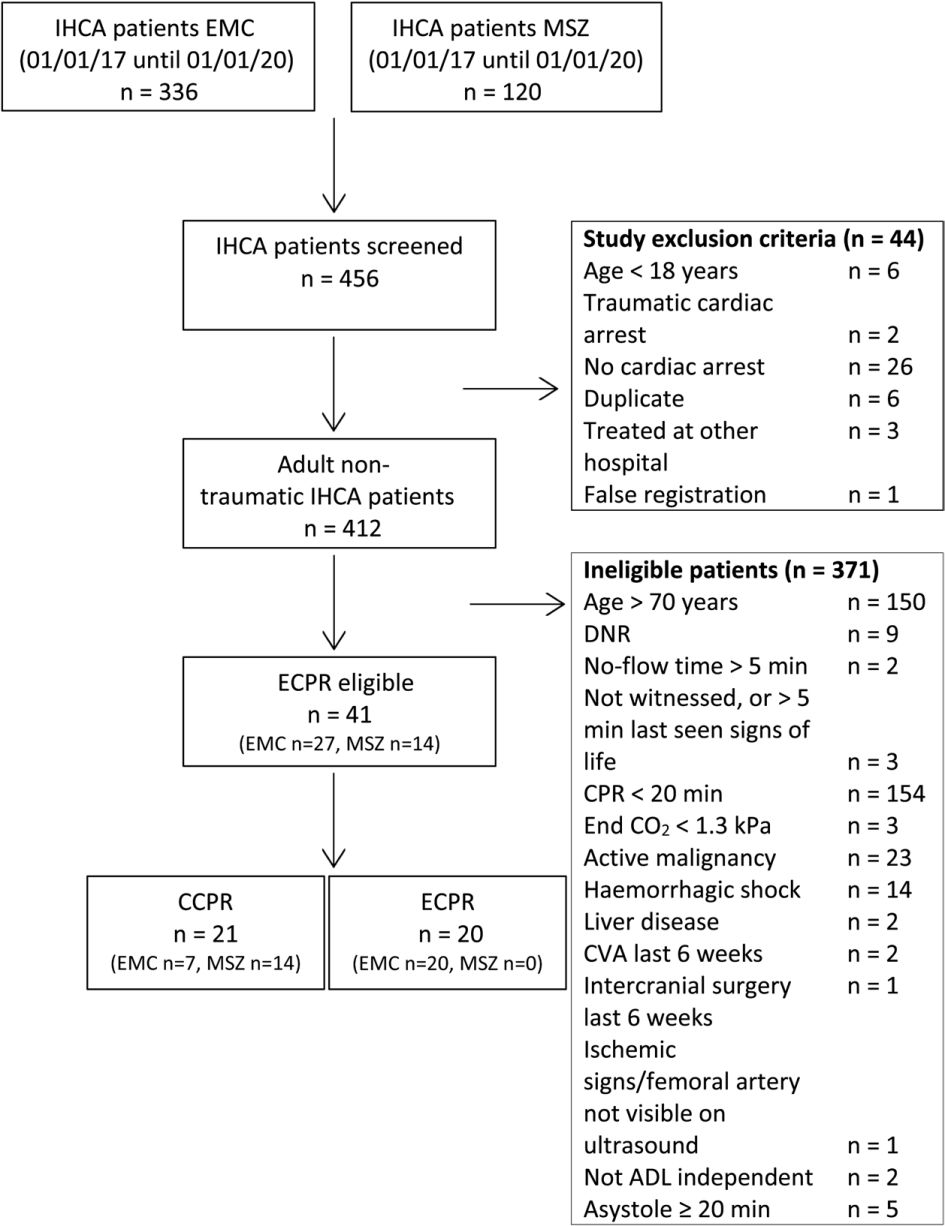
Flowchart with inclusion and exclusion of IHCA patients.

Figure 1 shows the number of included and excluded IHCA patients. In total, 456 IHCA patients were screened for eligibility. Of these 456 patients, 44 were excluded based on one of the study exclusion criteria. Of the remaining 412 patients who were screened for eligibility to ECPR, 41 (10.0%) were eligible to ECPR. Of these, 21 (51.2%) received CCPR and 20 (48.8%) received ECPR. Of the patients eligible to ECPR, 14 (34.1%) were treated in the hospital without ECPR facilities. Of the 27 patients eligible to ECPR in the hospital with ECPR facilities, 20 (74.1%) were actually treated with ECPR.

### Secondary outcomes: baseline characteristics of IHCA patients

Baseline characteristics of the IHCA patients are shown in Table 1. The median age was 59 years (IQR: 51–65) and 73.2% of the patients were male. The median no-flow time was 0.0 min in both the CCPR and ECPR group. The median low-flow duration was shorter in the CCPR group (29 min [23–31]) than in the ECPR group (45 min [39–63], (*P* < 0.01). The most common cause of cardiac arrest was coronary artery disease (24.4%). Additional characteristics can be found in Supplementary Material Table A, Supplemental digital content 1, http://links.lww.com/EJEM/A405.

**Table 1:** Baseline characteristics IHCA

	Total (N = 41)	CCPR (N = 21)	ECPR (N = 20)	*P*-value	Missing
Age	59.0 (51.0, 65.0)	60.0 (51.0, 65.0)	57.5 (51.3, 62.0)	0.449	0
Sex; male	30 (73.2%)	16 (76.2%)	14 (70.0%)	0.734	0
Arrest characteristics					
Witnessed arrest	41 (100.0%)	21 (100.0%)	20 (100.0%)		
Bystander life support	41 (100.0%)	21 (100.0%)	20 (100.0%)		
No flow time (min)	0.0 (0.0–0.0)	0.0 (0.0–1.0)	0.0 (0.0–0.0)	**0.011**	
Low flow time (min)	35.0 (26.8–45.0)	29.0 (23.0–31.0)	45.0 (39.0–62.5)	**<0.001**	3
CPR duration (no flow time + low-flow time, min)	35.0 (27.0–45.0)	30.0 (23.0–34.5)	45.0 (39.0–62.5)	**<0.001**	3
Mechanical CPR	6 (15.0%)	2 (9.5%)	4 (21.1%)	0.398	1
Cause of arrest					
Coronary artery disease	10 (24.4%)	5 (23.8%)	5 (25.0%)	1.000	
STEMI	8 (20.0%)	4 (20.0%)	4 (20.0%)	1.000	
Pulmonary embolism	7 (17.5%)	2 (9.5%)	5 (25.0%)	0.238	
Intoxication	1 (2.4%)	1 (4.8%)	0 (0.0%)	1.000	
Tamponade	4 (9.8%)	3 (14.3%)	1 (5.0%)	0.606	
Hypoxaemia	3 (7.3%)	1 (4.8%)	2 (10.0%)	0.606	
Electrolyte disorders	1 (2.4%)	1 (4.8%)	0 (0.0%)	1.000	
Arrhythmia	4 (9.8%)	1 (4.8%)	3 (15.0%)	0.605	
Other	2 (4.9%)	1 (4.8%)	1 (5.0%)	1.000	
Unknown	9 (22.0%)	6 (28.6%)	3 (15.0%)	0.451	
Primary cardiac rhythm					
Shockable rhythm	13 (31.7%)	6 (28.6%)	7 (35.0%)	0.744	
Non-shockable rhythm	26 (63.4%)	14 (66.7%)	12 (60.0%)	0.751	

CCPR, conventional CPR; CPR, cardiopulmonary resuscitation; ECPR, extracorporeal CPR; STEMI, ST-elevation myocardial infarction.

### Secondary outcomes: outcomes of IHCA patients

The outcomes of IHCA patients are shown in Table 2. Regarding the complications after cardiac arrest, AKI occurred significantly less often in CCPR than in ECPR (19.0% vs. 55.0% respectively, *P* = 0.03), the same applies to bleeding (4.8% vs. 40.0% respectively, *P* = 0.01).

**Table 2:** Outcomes IHCA patients

	Total (N = 41)	CCPR (N = 21)	ECPR (N = 20)	*P*-value
Complications				
Acute kidney injury	15 (36.6%)	4 (19.0%)	11 (55.0%)	**0.025**
CVA	2 (4.9%)	2 (9.5%)	0 (0.0%)	0.488
Re-arrest	8 (19.5%)	4 (19.0%)	4 (20.0%)	1.000
Infection	11 (26.8%)	6 (28.6%)	5 (25.0%)	1.000
Bleeding	9 (22.0%)	1 (4.8%)	8 (40.0%)	**0.009**
Post-anoxic brain injury	6 (14.6%)	2 (9.5%)	4 (20.0%)	0.410
Liver failure	4 (9.8%)	3 (14.3%)	1 (5.0%)	0.606
Delirium	5 (12.2%)	3 (14.3%)	2 (10.0%)	1.000
Other	9 (22.0%)	2 (9.5%)	7 (35.0%)	0.067
Outcomes				
ROSC	21 (51.2%)	10 (45.0%)	11 (55.0%)	0.758
Return of circulation	16 (39.0%)	NA	16 (80.0%)	-
ICU-survival	8 (25.0%)	5 (38.5%)	3 (15.8%)	0.219
Hospital survival	7 (17.1%)	4 (19.0%)	3 (15.0%)	1.000

CCPR, conventional CPR; CPR, cardiopulmonary resuscitation; CVA, cerebrovascular accident; ECPR, extracorporeal CPR; ROSC, return of spontaneous circulation; NA, not applicable.

Overall, the ICU survival was 25.0% and the hospital survival was 17.1% (19.0% in CCPR vs. 15.0% in ECPR patients, 4.0% survival difference 95% confidence interval −21.3 to 28.7%). In total, 57.7% of the patients eligible to ECPR with sustained ROSC regained consciousness. In the CCPR group, 58.8% of the patients died of ‘did not achieve ROSC’, which tended to differ from the ECPR group (23.5%, *P* = 0.08). Additional outcomes can be found in Supplementary Material Table C, Supplemental digital content 1, http://links.lww.com/EJEM/A405.

### Primary outcome OHCA patients

**Fig. 2:**
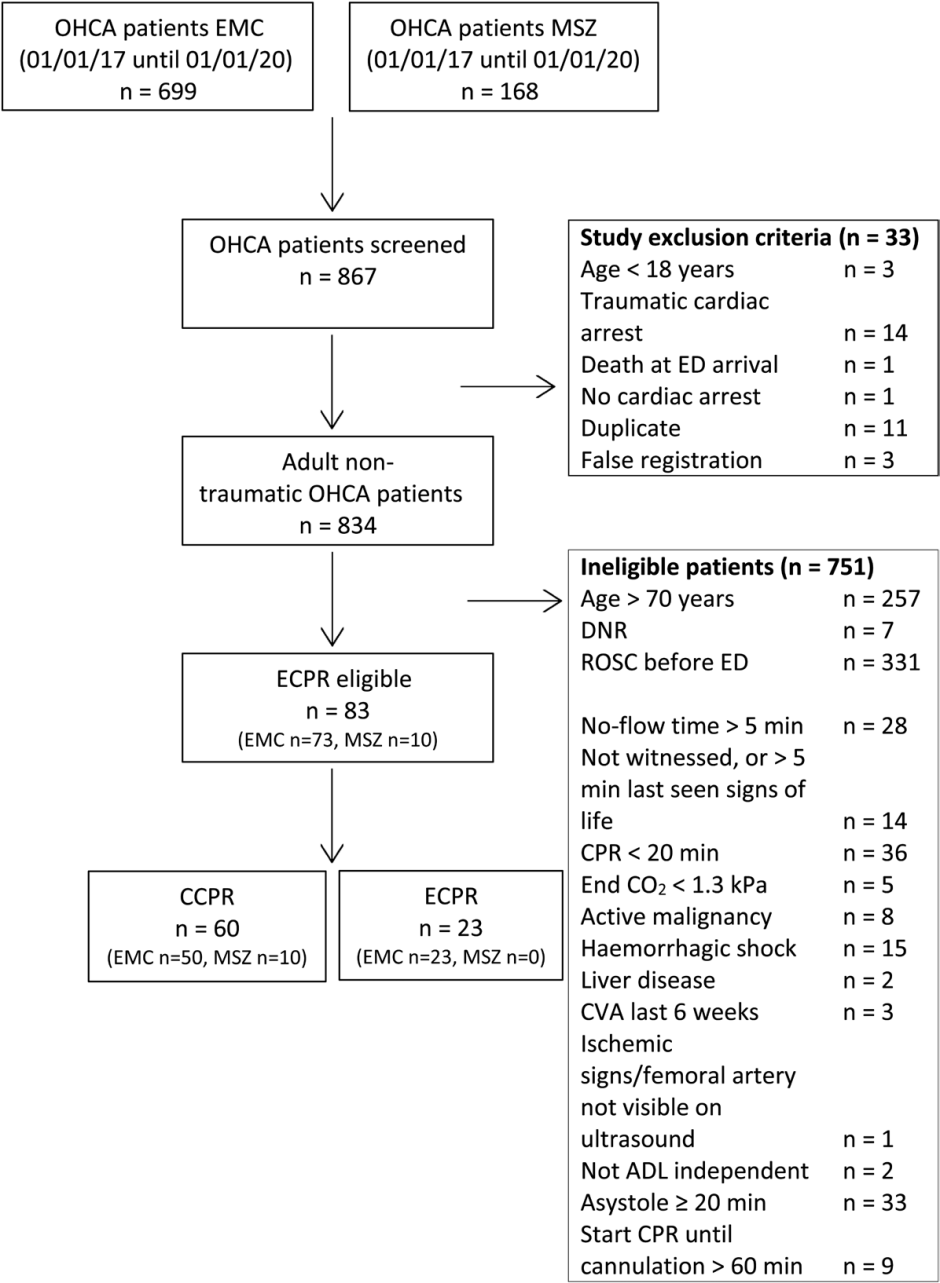
Flowchart with inclusion and exclusion of OHCA patients.

Figure 2 shows the number of included and excluded OHCA patients. In total, 867 OHCA patients were screened for eligibility of whom 83 (9.6%) were eligible to ECPR. Of these, 60 (72.3%) received CCPR and 23 (27.7%) received ECPR. Of the patients eligible to ECPR, 10 (12.0%) were treated in the hospital without ECPR facilities. Of the 73 patients eligible to ECPR in the hospital with ECPR facilities, 23 (31.5%) were actually treated with ECPR.

### Secondary outcomes: baseline characteristics of OHCA patients

Baseline characteristics of the OHCA patients are shown in Table 3. Additional characteristics can be found in Supplementary Material Table B, Supplemental digital content 1, http://links.lww.com/EJEM/A405. The median age was 56 years (IQR: 48–64) and 78.3% of the patients were male. Patients treated with CCPR were older than patients treated with ECPR, (60 vs. 46 years respectively, *P* < 0.01). The most common cause of cardiac arrest was coronary artery disease (50.6%).

**Table 3:** Baseline characteristics OHCA patients

	Total (N = 83)	CCPR (N = 60)	ECPR (N = 23)	*P*-value	Missing
Age	56.0 (47.5–63.5)	59.5 (51.8–65.0)	46.0 (33.5–51.5)	**<0.001**	
Sex: male	65 (78.3%)	46 (76.7%)	19 (82.6%)	0.767	
Arrest characteristics					
Witnessed arrest	76 (92.7%)	55 (93.2%)	21 (91.3%)	1.000	1
Bystander life support	69 (83.1%)	51 (85.0%)	18 (78.3%)	0.518	
No-flow time (min)	0.0 (0.0–2.0)	0.0 (0.0–2.0)	0.0 (0.0–2.0)	0.927	13
Low-flow time (min)	55.0 (40.0–65.0)	55.0 (35.5–61.0)	63.0 (46.0–80.0)	**0.032**	1
CPR duration (no-flow time + low-flow time, min)	56.0 (45.0–69.0)	55.5 (41.5–63.5)	60.0 (45.0–78.0)	0.196	14
Mechanical CPR	17 (20.5%)	5 (8.3%)	12 (52.2%)	**< 0.001**	
AED connected	51 (61.4%)	40 (66.7%)	11 (47.8%)	0.136	
Cause of arrest					
Coronary artery disease	42 (50.6%)	33 (55.0%)	9 (39.1%)	0.227	
STEMI	18 (21.7%)	11 (18.3%)	7 (30.4%)	0.247	
Pulmonary embolism	9 (10.8%)	4 (6.7%)	5 (21.7%)	0.107	
Intoxication	1 (1.2%)	1 (1.7%)	0 (0.0%)	1.000	
Tamponade	2 (2.4%)	2 (3.3%)	0 (0.0%)	1.000	
Hypoxaemia	1 (1.2%)	0 (0.0%)	1 (4.3%)	0.277	
Arrhtyhmia	6 (7.2%)	4 (6.7%)	2 (8.7%)	0.667	
Unknown	22 (26.5%)	16 (26.7%)	6 (26.1%)	1.000	
Primary cardiac rhythm					
Primary shockable rhythm	49 (59.0%)	36 (60.0%)	13 (56.5%)	0.807	
Primary non-shockable rhythm	32 (38.6%)	23 (38.3%)	9 (39.1%)	1.000	

AED, automatic external defibrillator; CCPR, conventional CPR; CPR, cardiopulmonary resuscitation; ECPR, extracorporeal CPR; STEMI, ST-elevation myocardial infarction.

### Secondary outcomes: outcomes of OHCA patients

The secondary outcomes of the OHCA patients are shown in Table 4. Post-anoxic brain injury occurred less often in CCPR patients than in ECPR patients (18.3% vs. 52.5% respectively, *P* < 0.01), the same applies to bleeding (5.0% vs. 26.1% respectively, *P* < 0.05). Overall, the ED survival was 50.6%, the ICU survival was 18.1% and the hospital survival was 15.7% (13.3% in CCPR vs. 21.7% in ECPR patients, 8.4% survival difference 95% confidence interval −30.3 to 10.2%). No significant difference was found in the ICU survival and hospital survival between CCPR and ECPR. In total, 23.8% of the patients eligible to ECPR with sustained ROSC regained consciousness, 29.2% in patients treated with CCPR and 16.7% in patients treated with ECPR (*P* = 0.47). In two patients eligible to ECPR, cannulation was started, but before the ECMO started these patients regained ROSC. Because ECMO was never started, these patients were included in the CCPR group. Additional outcomes can be found in Supplementary Material Table D, Supplemental digital content 1, http://links.lww.com/EJEM/A405.

**Table 4:** Outcomes OHCA patients

	Total (N = 83)	CCPR (N = 60)	ECPR (N = 23)	*P*-value	Missing
Complications					
Acute kidney injury	15 (18.1%)	10 (16.7%)	5 (21.7%)	0.751	
CVA	1 (1.2%)	1 (1.7%)	0 (0.0%)	1.000	
Re-arrest	7 (8.4%)	5 (8.3%)	2 (8.7%)	1.000	
Infection	7 (8.4%)	4 (6.7%)	3 (13.0%)	0.390	
Bleeding	9 (10.8%)	3 (5.0%)	6 (26.1%)	**0.012**	
Pulmonary embolism	0 (0.0%)	0 (0.0%)	0 (0.0%)		
Post-anoxic brain injury	23 (27.7%)	11 (18.3%)	12 (52.2%)	**0.005**	
Liver failure	4 (4.8%)	3 (5.0%)	1 (4.3%)	1.000	
Delirium	7 (8.4%)	4 (6.7%)	3 (13.0%)	0.390	
Other	7 (8.4%)	4 (6.7%)	3 (13.0%)	0.390	
Outcomes					
ROSC	37 (44.6%)	24 (40.0%)	13 (56.5%)	0.220	
Return of circulation	19 (22.9%)	NA	19 (82.6%)	-	
Emergency department survival	42 (50.6%)	24 (40.0%)	18 (78.3%)	**0.003**	
ICU-survival	15 (18.1%)	9 (15.0%)	6 (26.1%)	0.338	
Hospital survival	13 (15.7%)	8 (13.3%)	5 (21.7%)	0.336	

CCPR, conventional CPR; CPR, cardiopulmonary resuscitation; CVA, cerebrovascular accident; ECPR, extracorporeal CPR; NA, not applicable; ROSC, return of spontaneous circulation.

## Discussion

This study shows an ECPR eligibility rate of 10.0% for IHCA, and 9.6% for OHCA patients. This is in line with the existing literature. Iwashita *et al*. [20] and Olander *et al*. [26] found an ECPR eligibility rate in IHCA patients of 5.5–12.0%. The ECPR eligibility rate in OHCA patients is comparable; around 10.2–10.9% [17–19]. However, Gould *et al* [27] showed a higher eligibility rate of 14.1%. In their study, only non-traumatic cardiac arrest patients were included, which might be a possible explanation. Previous studies show a high rate of AED use and short travel time to the hospitals in the Netherlands [22–25]. Despite this, our study showed no difference in the proportion of OHCA patients eligible to ECPR. We were not able to show why this proportion is equal. As we speculate, we found several potential reasons. First, it could be that the high number of AED use, will result in a higher number of ROSC pre-hospital and therefore no need for ECPR treatment. This lower need for ECPR treatment in combination with shorter travel times could result in an equal eligibility percentage. Second, as every country has their own specific protocols and treatment options, it might be that criteria for ECPR treatment could differ and less patients will be eligible in the Netherlands due to stricter criteria. Third, EMS personal has to think about the ability to treat patients with ECPR in order to leave the location of the arrest more quickly. Last, it might be that the cause of arrest is a more important factor in the eligibility than previously expected.

In IHCA, 48.8% of the patients eligible to ECPR were actually treated with ECPR. In OHCA, 27.7% of the ECPR patients were actually treated with ECPR. Choi *et al*. [15] found a much higher eligibility rate of 53% in OHCA patients, of these eligible patients only 0.04% were treated with ECPR. However, the selection criteria in this study were less specific than in the current study, which affects both the eligibility rate as well as the number of ECPR-treated patients. In our study, a high rate of patients eligible to ECPR treated in the hospital with ECPR facilities were not treated with ECPR. Given the retrospective design of this study, it was unclear why these patients were not treated with ECPR.

No statistically significant difference in-hospital survival for patients eligible to ECPR treated with CCPR compared to patients treated with ECPR was found. Choi *et al*. [15] also showed no statistically significant difference regarding survival and neurologically favourable outcome in their propensity-matched cohort comparing CCPR- and ECPR-treated patients. Recently, several randomised controlled trials were performed to compare CCPR to ECPR in OHCA patients [28–30]. Two of them had to be terminated before the end of the study; Yannopoulos *et al*. [28] had to stop due to superiority of ECPR and Belohlavek *et al*. [29] had to stop due to futility. Suverein *et al*. [30] completed their study and they found no significant difference in outcomes of CCPR- and ECPR-treated patients. The inconsistent outcomes of these randomised controlled trials show that multiple factors, such as patient selection, experience in performing ECPR, low-flow duration, and transport times to the hospital, will determine if ECPR could be beneficial in cardiac arrest treatment. Another topic of future research will be the use of pre-hospital ECPR, as the benefits of this pre-hospital initiation is still not clear [31–33].

### Limitations

This study has several limitations. First, although the groups were screened for eligibility before emergency department admission – suggesting a more homogenous patient group – confounding by indication may still be a factor in the decision to pursue CCPR or ECPR. However, small differences between groups that may have affected this decision were found. This may suggest that the confounding by indication effect is small or that we were not able to capture the relevant clinical characteristics that affected the treatment decision. One such factor was missing etCO_2_. Second, during the study period, the INCEPTION study took place at the EMC (2017–2020) [30]. Some patients were ECPR eligible but were allocated to the CCPR group because of this INCEPTION trial. This may have caused a decrease in effect size, as one patient would have been part of the CCPR group while they actually belonged to the ECPR group. Third, the reason why eligible patients were not always treated with ECPR was unclear, this could be an aim for future research as this could contribute to the eligibility criteria. Last, the sample sizes of some subgroups might have been too small to reveal significant differences.

This study, as well as previous research, shows that only a small group of cardiac arrest patients are eligible to ECPR. Moreover, the outcomes regarding survival and neurological performance after ECPR vary. This makes it difficult to make a recommendation regarding the implementation of ECPR. On the one hand, more clinical experience with ECPR and improved protocols could further enhance the opportunities presented by this technique. On the other hand, complications are not uncommon in ECPR and it is an expensive technique that only a small group can benefit from. Therefore, treatment of sudden cardiac arrest patients with ECPR will remain a topic of discussion in terms of cost-effectiveness and which patients will benefit the most from it.

### Conclusion

This retrospective study shows that around 10% of cardiac arrest patients are eligible to ECPR. Less than half of these patients eligible to ECPR were actually treated with ECPR in both IHCA and OHCA.

## Acknowledgements

### Conflicts of interest

There are no conflicts of interest.

## Supplementary Material


