# Supplementary material for: Eligibility of cardiac arrest patients for extracorporeal cardiopulmonary resuscitation and their clinical characteristics: a retrospective two-centre study
Source: Eur J Emerg Med. 2023 Oct 6;31(2):118–26. doi: 10.1097/MEJ.0000000000001092 (PMC10901221; doi:10.1097/MEJ.0000000000001092)
Supplement: Supplementary file 1 [file ejem-31-118-s001.pdf]

Supplementary Material

Table A. Additional characteristics of IHCA patients

Legend to Supplementary Table A

ICD = internal cardiac defibrillator, IABP = intra-arterial balloon pump, CABG = coronary artery bypass graft, LVAD = left ventricular assist device, ECMO = extracorporeal membrane oxygenator.

	Total (N=41)	CCPR (N=21)	ECPR (N=20)	p-value	Missing
**Medical history**					
Cardiac	20 (48.8%)	12 (57.1%)	8 (40.0%)	0.354	0
Neurologic	3 (7.3%)	2 (9.5%)	1 (5.0%)	1.000	0
Pulmonary	9 (22.0%)	5 (23.8%)	4 (20.0%)	1.000	0
Gastro-intestinal	10 (24.4%)	6 (28.6%)	4 (20.0%)	0.719	0
Oncology	6 (14.6%)	3 (14.3%)	3 (15.0%)	1.000	0
Other	19 (46.3%)	11 (52.4%)	8 (40.0%)	0.536	0
**Cardiac risk factors**					
Diabetes Mellitus	8 (20.0%)	4 (19.0%)	4 (21.1%)	1.000	1
Hypercholesterolemia	5 (12.5%)	3 (14.3%)	2 (10.5%)	1.000	1
Obesity	8 (20.0%)	6 (28.6%)	2 (10.5%)	0.241	1
Hypertension	17 (42.5%)	9 (42.9%)	8 (42.1%)	1.000	1
Smoking	11 (27.5%)	3 (14.3%)	8 (42.1%)	0.078	1
Family history	7 (17.5%)	5 (23.8%)	2 (10.5%)	0.412	1
Alcohol abuse	4 (10.0%)	2 (9.5%)	2 (10.5%)	1.000	1
**Cause of arrest**					
Tension pneumothorax	0 (0.0%)	0 (0.0%)	0 (0.0%)		
Hypovolemia	0 (0.0%)	0 (0.0%)	0 (0.0%)		
Hypothermia	0 (0.0%)	0 (0.0%)	0 (0.0%)		
Neurological disease	0 (0.0%)	0 (0.0%)	0 (0.0%)		
**Primary cardiac rhythm**					

Ventricular fibrillation	10 (24.4%)	5 (23.8%)	5 (25.0%)	1.000	
Ventricular tachycardia	3 (7.3%)	1 (4.8%)	2 (10.0%)	0.606	
Asystole	2 (5.3%)	0 (0.0%)	2 (11.1%)	0.218	3
Pulseless electical activity	21 (55.3%)	13 (65.0%)	8 (44.4%)	0.328	3
**Medication used during CPR**					
Epinephrine	35 (87.5%)	17 (81.0%)	18 (94.7%)	0.345	1
Amiodarone	17 (42.5%)	9 (42.9%)	8 (42.1%)	1.000	1
Magnesium	4 (10.0%)	1 (4.8%)	3 (15.8%)	0.331	1
**Laboratory values**					
pH	7.08 (6.95-7.27)	7.05 (6.95-7.18)	7.16 (6.96-7.29)	0.322	6
Sodium	138.5 (134.3-143.0)	139.0 (134.0-145.0)	138.0 (137.0-141.5)	0.939	
Potassium	4.7 (4.0-5.2)	4.8 (4.3-5.1)	4.3 (3.3-5.3)	0.248	4
Lactate	7.7 (4.5-10.8)	9.6 (6.2-10.7)	6.9 (4.4-10.3)	0.423	10
Hb	7.3 (5.6-8.9)	8.0 (5.6-9.5)	7.1 (5.8-7.7)	0.335	3
CKMB	7.8 (3.8-1930)	10.3 (5.6-55.9)	5.1 (2.3-10.7)	0.120	23
High sensitive TroponinT	69.0 (1.3-499.5)	1.9 (0.7-141.2)	134.0 (72.8-1175.3)	**0.041**	18
End tidal CO2 (mmHg)	13.5 (11.3-15.0)	10.5 (10.5-10.5)	15.0 (13.5-15.0)	0.157	36
**Treatment**					
Percutaneus Coronary Intervention	10 (24.4%)	5 (23.08%)	5 (25.0%)	1.000	
ICD	2 (4.9%)	0 (0.0%)	2 (10.0%)	0.232	
IABP	8 (19.5%)	2 (9.5%)	6 (30.0%)	0.130	
CABG	1 (2.4%)	0 (0.0%)	1 (5.0%)	0.488	
Impella	1 (2.4%)	0 (0.0%)	1 (5.0%)	0.488	
VA-ECMO	2 (4.9%)	2 (9.5%)	0 (0.0%)	0.488	
LVAD	2 (4.9%)	0 (0.0%)	2 (10.0%)	0.232	

Inotropic and vasopressor use	21 (51.2%)	6 (28.6%)	15 (75.0%)	**0.005**	

Table B. Additional characteristics of OHCA patients

Legend to Supplementary Table B

ICD = internal cardiac defibrillator, IABP = intra-arterial balloon pump, CABG = coronary artery bypass graft, LVAD = left ventricular assist device, ECMO = extracorporeal membrane oxygenator.

	**Total (N=83)**	**CCPR (N=60)**	**ECPR (N=23)**	**p-value**	**Missing**
**Medical history**					
Cardiac	26 (31.3%)	22 (36.7%)	4 (17.4%)	0.116	
Neurologic	8 (9.6%)	7 (11.7%)	1 (4.3%)	0.434	
Pulmonary	7 (8.4%)	5 (8.3%)	2 (8.7%)	1.000	
Gastro-intestinal	0 (0.0%)	0 (0.0%)	0 (0.0%)		
Oncology	5 (6.0%)	4 (6.7%)	1 (4.3%)	1.000	
Other	20 (24.1%)	15 (25.0%)	5 (21.7%)	1.000	
**Cardiac risk factors**					
Diabetes Mellitus	15 (18.1%)	13 (21.7%)	2 (8.7%)	0.216	
Hypercholesterolemia	8 (9.6%)	5 (8.3%)	3 (13.0%)	0.679	
Obesity	6 (7.2%)	5 (8.3%)	1 (4.3%)	1.000	
Hypertension	28 (33.7%)	24 (40.0%)	4 (17.4%)	0.070	
Smoking	17 (20.5%)	10 (16.7%)	7 (30.4%)	0.224	
Family History	10 (12.0%)	7 (11.7%)	3 (13.0%)	1.000	
Alcohol abuse	3 (3.6%)	0 (0.0%)	3 (13.0%)	**0.019**	
Drugs abuse	7 (8.4%)	4 (6.7%)	3 (13.0%)	0.390	
**Cause of arrest**					
Tension pneumothorax	0 (0.0%)	0 (0.0%)	0 (0.0%)		
Hypovolemia	0 (0.0%)	0 (0.0%)	0 (0.0%)		
Hypothermia	0 (0.0%)	0 (0.0%)	0 (0.0%)		
Electrolyte disorder	0 (0.0%)	0 (0.0%)	0 (0.0%)		

Neurologic	0 (0.0%)	0 (0.0%)	0 (0.0%)		
Other	0 (0.0%)	0 (0.0%)	0 (0.0%)		
**Primary cardiac rhythm**					
Ventricular fibrillation	46 (56.8%)	34 (57.6%)	12 (54.5%)	0.807	2
Ventricular tachycardia	1 (1.2%)	1 (1.7%)	0 (0.0%)	1.000	2
Asystole	4 (5.1%)	3 (5.4%)	1 (4.3%)	1.000	4
Pulseless electrical activity	24 (30.4%)	16 (28.6%)	8 (34.8%)	0.600	4
**Medication used during CPR**					
Epinephrine	83 (100.0%)	60 (100.0%)	23 (100.0%)		
Amiodarone	49 (59.0%)	37 (61.7%)	12 (52.2%)	0.463	
Magnesium	14 (16.9%)	9 (15.0%)	5 (21.7%)	0.518	
**Laboratory values**					
pH	6.91 (6.79-7.00)	6.92 (6.82-7.00)	6.82 (6.75-6.92)	**0.004**	2
Sodium	141.0 (139.0-143.0)	140.0 (138.5-143.0)	141.0 (139.0-142.0)	0.803	1
Potassium	4.0 (3.3-4.6)	3.8 (3.2-4.6)	4.2 (3.6-4.6)	0.297	1
Lactate	12.7 (10.3-15.0)	11.7 (9.5-14.0)	14.7 (12.2-19.5)	**0.006**	3
Hb	8.9 (8.1-9.4)	9.0 (8.2-9.5)	8.8 (7.9-9.2)	0.168	1
CKMB	5.6 (3.7-15.9)	8.8 (3.9-17.9)	4.5 (3.6-8.0)	0.211	29
High sensitive troponin T	66.0 (21.0-224.0)	67.5 (21.0-230.8)	63.0 (24.5-190.5)	0.933	26
End tidal CO2 (mmHg)	27.0 (15.0-30.0)	28.5 (19.5-30.8)	15.0 (14.3-29.3)	0.140	43
**Treatment**					
Percutaneous coronary intervention	22 (26.5%)	14 (23.3%)	8 (34.8%)	0.405	
ICD	2 (2.4%)	2 (3.3%)	0 (0.0%)	1.000	
IABP	12 (14.5%)	7 (11.7%)	5 (21.7%)	0.299	
CABG	0 (0.0%)	0 (0.0%)	0 (0.0%)		
Impella	2 (2.4%)	2 (3.3%)	0 (0.0%)	1.000	
VA-ECMO	3 (3.6%)	3 (5.0%)	0 (0.0%)	0.557	
LVAD	0 (0.0%)	0 (0.0%)	0 (0.0%)		

Inotropic and vasopressor use	44 (53.0%)	26 (43.3%)	18 (78.3%)	**0.006**	

Table C. Additional outcomes in IHCA patients

Legend to Supplementary Table C

CCPR = conventional CPR, ECPR = extracorporeal CPR, GCS = Glasgow coma scale, ROSC = return of spontaneous circulation.

Analyses in patients with return of circulation				
	**Total (N=26)**	**CCPR (N=10)**	**ECPR (N=16)**	**p-value**
Length of stay (days)	6.5 (3.0-14.0)	11.5 (7.7-14.0)	4.0 (2.0-11.5)	0.119
Regain of consciousness	15 (57.7%)	7 (70.0%)	8 (50.0%)	0.428
Maximum GCS	7.0 (3.0-15.0)	15.0 (4.0-15.0)	3.0 (3.0-15.0)	0.094
Analyses in non-survivors				
**Cause of death**	**Total (N=34)**	**CCPR (N=17)**	**ECPR (N=17)**	**p-value**
No ROSC	14 (41.2%)	10 (58.8%)	4 (23.5%)	0.080
Multi-organ failure	8 (23.5%)	3 (17.6%)	5 (29.4%)	0.688
Single organ failure	3 (8.8%)	1 (5.9%)	2 (11.8%)	1.000
Post anoxic brain injury	6 (17.6%)	1 (5.9%)	5 (29.4%)	0.175
Bleeding	1 (2.9%)	0 (0.0%)	1 (5.9%)	1.000
Other	1 (2.9%)	1 (5.9%)	0 (0.0%)	1.000
Unknown	1 (2.9%)	1 (5.9%)	0 (0.0%)	1.000

Table D. Additional outcomes in OHCA patients

Legend to Supplementary Table D

CCPR = conventional CPR, ECPR = extracorporeal CPR, GCS = Glasgow coma scale, ROSC = return of spontaneous circulation.

Analyses in patients with return of circulation					
	**Total (N=42)**	**CCPR (N=24)**	**ECPR (N=18)**	**p-value**	**Missing**
Length of stay (days)	2.0 (1.0-6.0)	3.0 (1.0-8.0)	1.5 (1.0-5.0)	0.661	1
Regain of consciousness	10 (23.8%)	7 (29.2%)	3 (16.7%)	0.473	
Maximum GCS	3 (3-10)	3 (3-12)	3 (3-10)	0.704	
Analyses in non-survivors					
	**Total (N=70)**	**CCPR (N=52)**	**ECPR (N=18)**	**p-value**	**Missing**
**Cause of death**					
No ROSC	39 (55.7%)	35 (67.3%)	4 (22.2%)	**0.002**	
Multi-organ failure	4 (5.7%)	2 (3.8%)	2 (11.1%)	0.271	
Single organ failure	6 (8.6%)	5 (9.6%)	1 (5.6%)	1.000	
Post anoxic brain injury	13 (18.6%)	6 (11.5%)	7 (38.9%)	**0.030**	
Bleeding	4 (5.7%)	1 (1.9%)	3 (16.7%)	**0.050**	
Other	3 (4.3%)	2 (3.8%)	1 (5.6%)	1.000	

Appendix A (methods continued).

Prehospital organization

In the Netherlands a national emergency number will be called in case of a cardiac arrest. They will instruct the bystanders in performing basic life support (BLS) and will text nearby bystanders with CPR education to perform the CPR and get the automatic external defibrillator (AED). In the meanwhile, the closest ambulance will be dispatched. In case this ambulance is not close by, the police or fire department will be alarmed to continue BLS until the ambulance arrives. In case of a cardiac arrest, two ambulances will be send to the patient. Every ambulance has a specialized nurse to start advanced life support (ALS). In case a medical doctor is needed, a helicopter will be send. The focus of the EMS system in the Netherlands is to have an ambulance at scene within 20 minutes. After initiation of ALS, the EMS personal work with protocols, when to transport patients to which hospitals in the neighbourhood.

Appendix B (methods continued).

ECPR routine process

In the Erasmus MC hospital, ECPR-trained intensivists are 24/7 available for consultation in case of possible ECPR (IHCA and OHCA). Possible ECPR candidates are then screened for ECPR eligibility according to the criteria as mentioned (inclusion- and exclusion criteria). However, other factors can influence the final decision to start the ECPR procedure. ECPR is routine practice in the Erasmus MC.

In the Maasstad Hospital – also a PCI centre - no ECPR facilities are available. Therefore, no screening for ECPR-eligibility is done in cardiac arrest patients.

Intensivists and other ECPR personnel of the Erasmus MC are frequently trained by one of the ECPR-specialized intensivists according to the local training protocol.

Appendix C (methods continued).

ECPR procedure

When patients are eligible for ECPR and no ROSC is achieved within 20 minutes of CPR, cannulation of ECMO starts as soon as possible. For IHCA patients cannulation will be done at the location of the cardiac arrest. All OHCA patients are cannulated at the Emergency Department. A 17-19 Fr arterial cannula and a 21-25 Fr venous cannula are placed in the femoral artery and vein (Gettinge group, Maquet HLS cannula). After cannula placement, the Cardiohelp System is connected (Gettinge group, Maquet Cardiopulmonary GmbH, Germany). Within six hours after ECPR placement, all patients receive an antegrade 6Fr cannula in the ipsilateral superficial femoral artery.
